# Effect of Dapagliflozin on Urine Metabolome in Patients with Type 2 Diabetes

**DOI:** 10.1210/clinem/dgab086

**Published:** 2021-02-16

**Authors:** Evdoxia Bletsa, Sebastien Filippas-Dekouan, Christina Kostara, Panagiotis Dafopoulos, Aikaterini Dimou, Eleni Pappa, Styliani Chasapi, Georgios Spyroulias, Anastasios Koutsovasilis, Eleni Bairaktari, Ele Ferrannini, Vasilis Tsimihodimos

**Affiliations:** 1 Third Internal Medicine Department, General Hospital of Nikaia, Athens, Greece; 2 Department of Internal Medicine, University of Ioannina, Ioannina, Greece; 3 Laboratory of Clinical Chemistry, University of Ioannina, Ioannina, Greece; 4 Department of Pharmacy, University of Patras, Rio, Greece; 5 CNR Institute of Clinical Physiology, Pisa, Italy

**Keywords:** branched chain amino acids, dapagliflozin, kidney, metabolomics, osmolytes

## Abstract

**Context:**

Inhibitors of sodium-glucose cotransporters-2 have cardio- and renoprotective properties. However, the underlying mechanisms remain indeterminate.

**Objective:**

To evaluate the effect of dapagliflozin on renal metabolism assessed by urine metabolome analysis in patients with type 2 diabetes.

**Design:**

Prospective cohort study.

**Setting:**

Outpatient diabetes clinic of a tertiary academic center.

**Patients:**

Eighty patients with hemoglobin A1c > 7% on metformin monotherapy were prospectively enrolled.

**Intervention:**

Fifty patients were treated with dapagliflozin for 3 months. To exclude that the changes observed in urine metabolome were merely the result of the improvement in glycemia, 30 patients treated with insulin degludec were used for comparison.

**Main Outcome Measure:**

Changes in urine metabolic profile before and after the administration of dapagliflozin and insulin degludec were assessed by proton-nuclear magnetic resonance spectroscopy.

**Results:**

In multivariate analysis urine metabolome was significantly altered by dapagliflozin (R^2^X = 0.819, R^2^Y = 0.627, Q^2^Y = 0.362, and coefficient of variation analysis of variance, *P* < 0.001) but not insulin. After dapagliflozin, the urine concentrations of ketone bodies, lactate, branched chain amino acids (*P* < 0.001), betaine, myo-inositol (*P* < 0001), and N-methylhydantoin (*P* < 0.005) were significantly increased. Additionally, the urine levels of alanine, creatine, sarcosine, and citrate were also increased (*P* < 0001, *P* <0.0001, and *P* <0.0005, respectively) whereas anserine decreased (*P* < 0005).

**Conclusions:**

Dapagliflozin significantly affects urine metabolome in patients with type 2 diabetes in a glucose lowering-independent way. Most of the observed changes can be considered beneficial and may contribute to the renoprotective properties of dapagliflozin.

Inhibitors of sodium glucose co-transporters-2 (SGLT-2) are a relatively new class of antidiabetic agents that exert their glucose-lowering action by promoting glucosuria even at physiological plasma glucose concentrations. In addition to their antihyperglycemic effect, these compounds decrease body weight and blood pressure and have a beneficial impact on uric acid homeostasis and arterial stiffness. Large clinical trials have shown that these drugs significantly reduce the incidence of diabetic nephropathy and the rate of hospitalizations for heart failure (1-4) and, in selected populations, decrease total and cardiovascular mortality. As a consequence, SGLT-2 inhibitors are now considered as the drugs of choice for patients with very high or high cardiovascular risk and/or diabetic nephropathy (5). However, what remains largely unknown is the mechanisms that underlie these beneficial effects. Although the change in the conventional cardiovascular or renal risk factors as just described can be an answer, the very early emergence of the cardiovascular benefit makes this possibility unlikely. Novel mechanisms that have been proposed during the previous years point at the effect of these drugs on energy metabolism either locally (myocardial or renal metabolism) or systemically (6,7).

Among the new approaches that have been recently developed to improve understanding within molecular biology, metabolomics—the qualitative and quantitative assessment of metabolites in biological matrices (body fluids, tissues, cells, etc)—is now a widely utilized methodology. Simultaneous profiling of multiple metabolites in a biological system, called metabolic phenotyping, combined with multivariate statistical analysis, has been successfully used for biomarker discovery, understanding disease processes, and exploring the involvement of nongenetic influences, such as gut microflora. Metabolomics has been extensively applied for the understanding of the metabolic footprint of diabetes, to reveal novel insights into the biochemical consequences of the disease, and to identify biomarker panels or specific molecules, such as branched chain amino acids (BCAAs) (8), alpha-hydroxybutyrate (9), and 2-aminoadipic acid (10), related to altered metabolic processes of major fuel classes (11).

Previous metabolomic studies in patients receiving SGLT-2 inhibitors have shown a shift in energy metabolism toward the use of substrates other than glucose (such as ketone bodies or amino acids) (12) as well as an improvement in mitochondrial function (13) following the administration of empagliflozin or dapagliflozin, respectively. These studies either used plasma metabolomics only (12) or performed urine metabolomics in patients with established diabetic nephropathy (13). In the present study we explored the effect of dapagliflozin on proton-nuclear magnetic resonance (^1^H-NMR)-based urine metabolomic signature in patients with type 2 diabetes of early onset who had normal renal function at baseline. For this reason, we assessed the urine metabolic profile of patients with type 2 diabetes before and after the administration of dapagliflozin and insulin degludec using ^1^H-NMR spectroscopy.

## Materials and Methods

### Patients

Fifty patients with type 2 diabetes on metformin monotherapy (at least 2000 mg daily or maximum tolerated dose) with hemoglobin A1c (HbA1c) > 7% were included between June 2016 and March 2018 (dapagliflozin group). All patients were given dapagliflozin 10 mg daily for 3 months. Patient with preexisting kidney disease [estimated glomerular filtration rate (eGFR) < 60 mL^.^min^−1.^1.73m^−2^), history of treated hypertension, established cardiovascular disease or heart failure were excluded. Patient were asked to keep their dietary habits as constant as possible during the study. In addition, during this period the dose of concomitant medications (lipid-lowering drugs, antiplatelet agents, etc) was kept stable. Study participants were followed up at monthly intervals for safety reasons. Since there are limited data on the reference values for the various urine low molecular weight metabolites, we included 53 healthy age- and sex-matched individuals to serve as controls. In addition, to exclude that the effects of dapagliflozin on urine metabolome represent nonspecific consequences of the correction of hyperglycemia, we compared them with those observed in a group of 30 patients with type 2 diabetes and HbA_1c_ > 7% on metformin monotherapy that were treated with insulin degludec for 3 months (insulin group). Degludec was administered in 1 daily dose every evening, and it was titrated every 3 days to a target of fasting glucose 100 to 120 mg/dL. All study subjects provided written informed consent prior to any trial procedure. The trial protocol was approved by the Greek Food and Drug Administration and the Scientific Committee of the University Hospital of Ioannina. The study was registered with ClinicalTrials.gov (identifier: NCT02798757).

### Analytical methods

Blood and urine samples were collected in the morning, after an overnight fast, at baseline and at the end of the active treatment period. Serum was separated by centrifugation at 1500 *g* for 15 min and an aliquot was stored at −80°C until nuclear magnetic resonance (NMR) analysis. Hematological and biochemical parameters were measured by standard laboratory methods. HbA1c were determined by a HPLC Variant II analyzer (Bio-Rad Laboratories, Munich, Germany). Urine samples were centrifuged at 1000 g for 10min and stored at −80°C until NMR analysis. Biochemical parameters were measured directly by standard laboratory methods. Urinary α1-microglobulin and immunoglobulin G levels were measured by immunonephelometry on a BN ProSpec System (Siemens, Marburg, Germany).

### ^1^H NMR spectroscopy

Urine samples were thawed and 400 µL were mixed with 200 µL of phosphate buffer and centrifuged at 8000 rpm. Five-hundred µL of the supernatant was mixed with 50 µL of sodium-3-trimethylsilyl-(2,2,3,3,-2H4)-propionate (TSP) in D_2_O to a final concentration 0.456 mmol/L and transferred to 5 mm tubes for the NMR measurements. Serum samples were deproteinized before the previously mentioned procedure using the centrifugal filter devices Amicon Ultra-2mL, 3-kDa cutoff (Merck KGaA, Darmstadt, Germany).

### NMR spectra acquisition and data processing

^1^H-NMR spectra were recorded at 300K on an Avance III 700 MHz spectrometer equipped with a cryogenically cooled gradient probe (Bruker BioSpin GmbH). A standard nuclear overhauser enhancement spectroscopy pulse sequence with a relaxation delay of 4 s and a mixing time of 0.01 s for serum, and 0.015 s for urine was used to suppress the water signal. For each spectrum, a total of 128 scans for serum and 64 for urine were collected into 64K data points over a spectral width of 14.9995 Hz with an acquisition time of 3.5 and 3.12 s, respectively. Free-induction decays were multiplied with an exponential line broadening function of 0.3 Hz prior to Fourier transformation. NMR spectra were manually corrected for phase and baseline distortions (Topspin 4.0.6, Bruker Biospin, Rheinstetten, Germany) and referenced to TSP (δ1H 0.0).

Spectral intensities were scaled to the total intensity and reduced to equidistant integrated regions of 0.04 ppm. Spectral region related to residual water and urea (4.38 and 6.30 ppm, respectively) was excluded. Before the multivariate analysis, the data set was preprocessed using the interval correlation optimized shifting (Icoshift) algorithm (14) (Matlab, version 8.5, MathWorks, Natick, MA, USA) to minimize spectral peak shift due to residual pH differences within samples and exported to the SIMCA-P+ 15 software package (Umetrics, Umea, Sweden), centered and Pareto scaled for multivariate statistical analysis.

### Targeted metabolite profiling

Metabolite identification was performed according to Chenomx NMR Suite 8.4 profiler (Chenomx Edmonton, Canada), the available databases such as the Human Metabolome Database (http://www.hmdb.ca), Biological Magnetic Resonance Data Bank (http://www.bmrb.wisc.edu), J-res 2D experiments, and the existing NMR-based metabolomics literature. Τhe 700 MHz library of the Chenomx NMR Suite software was used for the metabolites quantification, urinary creatinine was used as an internal reference, and values were expressed as µmoles of metabolite per mmol (μΜ/mM) of creatinine. For serum samples, an internal standard of known concentration (TSP) was used for the obtained quantitative values of metabolites concentration, which were presented in micromoles per liter (μM).

### Calculations

Body mass index was calculated as weight (kg) divided by height squared (m^2^). Glomerular filtration rate was estimated from serum creatinine, using the modification of diet in renal disease study equation. Fractional excretion of uric acid and electrolytes was calculated from the standard formula:


$$\begin{array}{l} \%FE\chi =(U\chi \times \;Scr/S\chi \times Ucr)\times \;100\% \end{array}$$

where Sχ and Uχ represent the serum and urine concentrations of electrolyte χ, and Scr and Ucr serum and urine concentrations of creatinine, respectively.

### Statistical analysis

Statistical analysis was performed using the SPSS v20.0 statistical package. Check for normality was done using the Kolmogorov-Smirnov test. The mean concentration of the various metabolic parameters before and after the administration of dapagliflozin was compared using Student’s paired *t*-test for normally distributed values and Wilcoxon matched pairs test for values deviating from normal distribution. The Bonferroni correction was applied to account for multiple comparisons. Two-way repeated measures analysis of variance was used for the comparison of the effects of dapagliflozin and insulin degludec on the concentrations of the various metabolites. The correlations between these changes were assessed using linear regression analysis after log-transformation of the values that did not follow normal distribution. Independent *t*-test was used for the comparison of the percentage changes in the concentrations of urine metabolites in dapagliflozin and insulin groups.

### Multivariate statistical analysis

Principal component analysis (PCA) and orthogonal projections to latent structures discriminant analysis (OPLS-DA) were used to construct pattern recognition models. PCA was used to obtain a general overview on samples and highlight possible clusters, trends, or outliers followed by OPLS-DA analysis to eliminate the uncorrelated systematic variation and describe the maximum separation based on class membership. The results of OPLS-DA analysis displayed by scores (detection observations lying outside the 0.95 Hotteling’s T2 ellipse, grouping trend, or separation) and loading coefficient plots (contribution of NMR spectral regions or variables, corresponding to metabolites. to the grouping trend or separation seen in the scores plot). OPLS-DA models were assessed by goodness-of-fit parameters R^2^ (R^2^X and R^2^Y) and Q^2^, related, respectively, to the explained and predicted variance. Cross-validated coefficient of variation analysis of variance (CV-ANOVA) and permutation tests were used to assess the significance and validity of the resulting OPLS-DA models, respectively (15). Finally, model validation was performed by constructing new OPLS-DA models with 80% of randomly selected samples considered as a training set, whereas the remaining 20% of samples, named as a test set, was used to predict their class membership.

## Results

The aim of the present study was to assess the urine metabolic profile before and after the administration of dapagliflozin in 50 patients with type 2 diabetes on metformin monotherapy using ^1^H-NMR spectroscopy (dapagliflozin group). For comparison, we also determined the urine metabolic profile of 53 healthy age- and sex-matched individuals (controls) and in 30 patients before and after the administration of insulin degludec (insulin group). Of the 50 patients treated with dapagliflozin, 2 stopped the investigational product prematurely. The discontinuation was not due to an adverse event and both patients did not withdraw their consent. As a consequence, they were included in the intention to treat analysis. With the exception of these 2 patients, treatment compliance, as assessed by tablet counting, was 100%.

The baseline demographic and clinical characteristics of the study participants are summarized in Table 1. Most patients were male, their mean age was 60 years, and they had a relatively short history of type 2 diabetes. As expected, many of them were overweight or obese. There was a slight imbalance in the baseline characteristics of patients in the dapagliflozin and insulin groups. For example, patients in the dapagliflozin group had blood pressure readings consistent with grade 1 hypertension while blood pressure values in the insulin group were similar to those of controls. In addition, while patients in the dapagliflozin group had higher eGFR values compared to control group, mean eGFR in the insulin group was lower than that in the control and dapagliflozin groups. (Table 2).

**Table 1.** Demographic and clinical characteristics of the study population

	Controls	Dapagliflozin	Insulin
N	53	50	30
Males/females (%)	60/40	60/40	50/50
Age (years)	59 ± 6	60 ± 8.5	63 ± 7.7
Diabetes duration (years)	—	4.2 ± 1.4	5.7 ± 2.3
Body mass index (kg/m^2^)	24.8 ± 3.2	32.1 ± 5.2	29.7 ± 1.1
Smokers (%)	20	17	20
Systolic blood pressure (mmHg)	120 ± 12	150 ± 18	137 ± 16
Diastolic blood pressure (mmHg)	78 ± 10	93 ± 13	79 ± 10

**Table 2.** Clinical characteristics and serum and urine biochemical parameters before and 3 months after dapagliflozin and insulin degludec

	Controls	Dapagliflozin			Insulin		
		Before	After	*P*-value	Before	After	*P*-value
Clinical parameters							
Weight (kg)	72.2 ± 9.1	92.8 ± 14.8**	91.1 ± 12.2**	0.001	87.8 ± 9.2** ^§^	88.1 ± 8.9** ^§^	NS
Body mass index (kg/m^2^)	24.8 ± 3.2	32.1 ± 5.2**	31.8 ± 5.6**	0.001	29.7 ± 1.1** ^§^	29.9 ± 1.3** ^§^	NS
Systolic blood pressure (mmHg)	120 ± 12	150 ± 18**	138 ± 15*	0.001	137 ± 16^§^	136 ± 13	NS
Diastolic blood pressure (mmHg)	78 ± 10	93 ± 13*	87 ± 15*	0.001	79 ± 10^§^	80 ± 8.3^§^	NS
Heart rate (bpm)	76 ± 12	80 ± 15	79 ± 12	NS	80 ± 8	78 ± 4	NS
Serum biochemical parameters							
Blood glucose (mmol/L)	4.94 ± 0.78	9.32 ± 3.05**	7.88 ± 2.00**	0.001	8.82 ± 1.39**	7.55 ± 1.44**	0.001
HbA1c (mmol/mol)	32.2 ± 7.9	65 ± 9.2**	57.4 ± 10.2**	0.001	66.1 ± 9.7**	54.1 ± 8.7**	0.001
HbA1c (%)	5.1 ± 0.7	8.1 ± 0.9**	7.4 ± 0.9**	0.001	8.2 ± 0.9**	7.1 ± 0.8**	0.001
Insulin (pmol/L)	—	15.9 ± 8.8	13.2 ± 11.8	NS			
Urea (mmol/L)	12.89 ± 2.61	12.89 ± 3.46	13.42 ± 4.25	NS	16.78 ± 6.43	16.06 ± 5.35	NS
Creatinine (μmol/L)	86.63 ± 14.14	74.26 ± 15.03*	76.91 ± 15.03*	0.005	97.24 ± 26.52^§^	97.24 ± 26.52^§^	NS
eGFR (mL^.^min^−1.^1.73m^−2^)	80.2 ± 12.4	89.3 ± 14.9*	85.7 ± 16.1*	0.005	72.4 ± 8.1^§^	71.8 ± 7.4^§^	NS
Total cholesterol (mmol/L)	5.8 ± 1.04	4.87 ± 1.29	4.71 ± 0.96	NS	4.84 ± 1.06	4.66 ± 0.93	NS
Triglycerides (mmol/L)	1.33 ± 0.66	1.84 ± 0.96	1.73 ± 0.67	NS	1.91 ± 1.20	1.71 ± 1.27	NS
HDL-cholesterol (mmol/L)	1.5 ± 0.34	1.14 ± 0.23	1.17 ± 0.26	NS	1.22 ± 0.26	1.22 ± 0.34	NS
LDL-cholesterol (mmol/L)	3.7 ± 0.93	2.85 ± 1.06	2.72 ± 0.88	NS	2.75 ± 0.88	2.69 ± 0.7	NS
Uric acid (mmol/L)	0.32 ± 0.07	0.32 ± 0.09	0.28 ± 0.07	0.001	0.32 ± 0.12	0.3 ± 0.07	NS
Sodium (mmol/L)	140 ± 2	138 ± 2	139 ± 2	NS	137.5 ± 2.4	138.7 ± 1.9	NS
Potassium (mmol/L)	4.5 ± 0.3	4.5 ± 0.4	4.5 ± 0.4	NS	4.5 ± 0.5	4.6 ± 0.4	NS
Calcium (mmol/L)	2.38 ± 0.35	2.38 ± 0.1	2.4 ± 0.1	NS	2.4 ± 0.07	2.38 ± 0.1	NS
Magnesium (mmol/L)	1.8 ± 0.1	1.7 ± 0.2	1.8 ± 0.3	NS	1.5 ± 0.2	1.5 ± 0.2	NS
Phosphorus (mmol/L)	1.29 ± 0.19	1.13 ± 0.19	1.23 ± 0.16	0.005	1 ± 0.16	1.03 ± 0.23	NS
Chloride (mmol/L)	100.9 ± 2.3	101.9 ± 1.9	101.9 ± 2.5	NS	101. 6 ± 3.2	102.1 ± 3.7	NS
Intact parathyroid hormone (ng/L)	—	37.7 ± 14.8	39.3 ± 15.2	NS	42.5 ± 11.3	45.3 ± 12.4	NS
Urine biochemical parameters							
Microalbumin (mg/g creatinine)	—	17.7 ± 16.6	14.4 ± 11.6	0.005	22 ± 18	25 ± 20	NS
FE uric acid (%)	—	7.2 ± 2.4	7.8 ± 3.4	0.005	8.8 ± 6.6	7.2 ± 3.9	NS
FE sodium (%)	—	0.62 ± 0.43	0.56 ± 0.31	NS	0.7 ± 0.4	0.9 ± 0.7	NS
FE potassium (%)	—	9.3 ± 4.2	12.5 ± 8.5	NS	10.5 ± 4.7	12.5 ± 8.6	NS
FE calcium (%)	—	0.9 ± 0.7	1.1 ± 0.9	NS	0.8 ± 0.6	1.1 ± 0.8	NS
FE magnesium (%)	—	2.2 ± 1.2	2.3 ± 1.2	NS	3.2 ± 1.6	4.4 ± 3.1	NS
FE phosphate (%)	—	11.7 ± 4.4	10.9 ± 5.3	0.005	13.3 ± 4.5	14.2 ± 6.2	NS
FE chloride (%)		1.1 ± 0.6	1.1 ± 0.5	NS	1.1 ± 0.6	1.3 ± 0.5	NS

**P < 0.001 and *P < 0.01 compared to control group; ^§§^*P* < 0.001 and ^§^*P* < 0.01 compared to dapagliflozin group at the same time-point (baseline or post-treatment)

Abbreviations: FE, fractional excretion; HDL, high-density lipoprotein; LDL, low-density lipoprotein.

Dapagliflozin significantly reduced body mass index, blood pressure values, and glycemic indices without significantly affecting the serum levels of insulin (Table 2). Serum creatinine showed a small but significant increase after dapagliflozin administration (by 2.5 μmol/L, *P* = 0.005). The drug had no consistent effect on serum lipids. On the other hand, dapagliflozin induced a significant decrease in the serum concentrations of uric acid (from 0.32 ± 0.09 to 0.28 ± 0.07 mmol/L, *P* = 0.001) and a small but significant elevation in the levels of serum phosphate (from 1.13 ± 0.19 to 1.23 ± 0.16 mmol/L, *P* = 0.005). These latter changes where accompanied by reciprocal changes in the renal fractional excretion values of these compounds (Table 2). Finally, although the patients had only marginal degree of albuminuria at baseline, dapagliflozin significantly reduced urine albumin at 3 months (from 17.7 ± 16.6 to 14.4 ± 11.6 mg/g creatinine, *P* = 0.005). On the other hand, most of the patients had urine α-1-microglobulin and immunoglobulin G concentrations below the detection limit of the method, and this did not change with dapagliflozin administration (data not shown). Insulin degludec, apart from its effect on HbA1c and fasting glucose levels, had no meaningful effect on plasma and urine conventional metabolic parameters (Table 2).

Supplementary Figure 1 (16) shows a representative ^1^H-NMR spectrum of urine from a patient with T2D at baseline. The main constituents of the urine spectrum were creatinine, hippurate, citrate, glycine, trimethylamine-*N*-oxide, dimethylamine, lactate, 3-hydroxybutyrate, and amino acids such as alanine, phenylalanine, tyrosine, and the BCAAs valine, leucine, and isoleucine (Supplementary Table 1 (16)). For the accurate assessment of the effect of dapagliflozin on urine metabolome, the metabolic profile of urine as recorded by ^1^H-NMR spectroscopy was then analyzed by multivariate data analysis (untargeted analysis) as well as by quantification of selected metabolite signals (targeted analysis).

**Figure 1.**
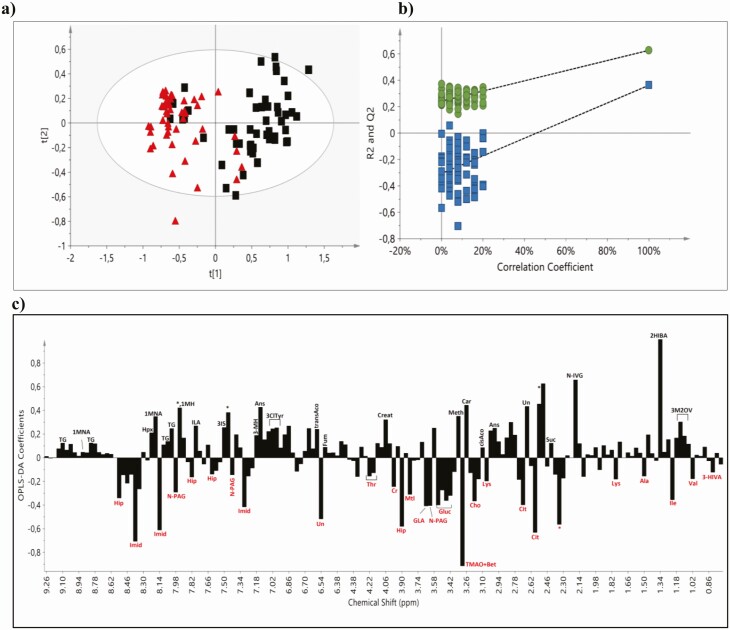
**(A)** OPLS-DA and validation **(B)**of the corresponding OPLS-DA models via permutations tests (n = 100) of the urine metabolic data from patients with type 2 diabetes mellitus before (black squares) and 3 months after (red triangles) administration of dapagliflozin. The regression line of Q^2^ (goodness of prediction, blue squares) with intercept at −0.306 and R^2^ (goodness of fit, green circles) regression line intercept at 0.242 indicate that the resulting OPLS-DA model has higher R^2^ (goodness of fit) and Q^2^ (goodness of prediction) values in the validation test than the permutated models generated. **(C)** The corresponding OPLS-DA regression coefficient plot of the urine metabolites, upper (black): higher before and down (red): higher after treatment. Abbreviations: 1MH, 1-methylhistidine; 1MNA, 1-methylnicotinamide; 2HIBA, 2-hydroxyisobutyrate; 3ClTyr, 3-chlorotyrosine; 3HIVA, 3-hydroxyisovalerate; 3IS, 3-indoxylsulfate; 3M2OV, 3-methyl-2-oxovalerate; 3MH, 3-methylhistidine; Ala, alanine; Ans, anserine; Bet, betaine; Car, carnitine; Cho, choline; cisAco, cis-aconitate; Cit, citrate; Cr, creatine; Creat, creatinine; Fum, fumarate; GLA, gluconate; Gluc, glucose; Hip, hippurate; Hpx, hypoxanthine; ILA, indole-3-lactate; Ile, isoleucine; Imid, imidazole; Lys, lysine; Mtl, mannitol; Meth, methanol; N-IVG, N-isovaleroylglycine; N-PAG, N-phenylacetylglycine; Suc, succinate; TG, trigonelline; Thr, threonine; TMAO, trimethylamine N-oxide; transAco, trans-aconitate; Val, valine; *, drug metabolites; Un, unknown.

For the untargeted analysis the data set consisted of the 1-dimensional ^1^H-NMR spectra of the urine derived from all patients before and after the administration of dapagliflozin and insulin degludec. PCA was initially applied to obtain a general overview (Supplementary Figure 2 (16)). No remarkable outliers were observed in the scores plot whereas a separation trend was appeared between diabetic patients before and after treatment with dapagliflozin. In the OPLS-DA scores plot of the untargeted metabolic profile (Fig. 1A), the groups before and after the administration of dapagliflozin were well separated with a small degree of overlap, with the samples of diabetic patients before treatment placed on the right half of the plot, and those after treatment, on the left half. The separation between the 2 groups assessed by the following quality parameters of the resulting OPLS-DA model: R^2^X = 0.819, R^2^Y = 0.627, and Q^2^Y = 0.362, and the CV-ANOVA *P*-value was <0.001. As shown in Figure 1B, the OPLS-DA model had a R^2^Y intercept of 0.242 and a Q^2^Y intercept value of −0.306, indicating that the resulting statistical model is valid.

The OPLS-DA loading coefficient plot, which depicts the main spectral regions contributing to group discrimination, yielded a rather large number of metabolites (Fig. 1C). In addition to marked glycosuria, posttreatment samples presented higher levels of hippurate, citrate, trimethylamine N-oxide (TMAO) and betaine, isoleucine, choline, gluconate, and N-phenylacetylglycine, as well as lower levels of 2-hydroxyisobutyrate, carnitine, N-isovaleroylglycine, trigonelline, methanol, and anserine as compared to those before treatment. Higher excretion of mannitol, creatine, and valine and lower excretion of 3-methyl-2-oxovalerate, 3-methylhistidine, trans-aconitate, and hypoxanthine made a smaller contribution to the group separation.

To test the reliability of the OPLS-DA model between diabetic patients before and after treatment with dapagliflozin, validation was carried out. The corresponding training (40 patients before/40 patients after treatment) and test (10 patients before/10 patients after treatment) sets were randomly selected, and the validation was repeated 3 times with a new random selection of equally numbered sets each time. The average classification rate was 86.67% for patients before treatment (first repeat: 9 out of 10; second repeat: 9 out of 10; and third repeat: 8 out of 10) and 90% for those after treatment (first repeat: 8 out of 10; second repeat: 9 out of 10; and third repeat: 10 out of 10) (Supplemenatry Figure 3 (16)).

PCA was applied and no distinct grouping was identified between the patients before and after the administration of insulin degludec in the scores plot (Supplementary Figure 4A) (16). OPLS-DA scores also did not show significant separation between the 2 groups (Supplementary Figure 4B) (16) with low goodness-of-fit parameters (R^2^X = 0.937, R^2^Y = 0.414, and Q^2^Y = -0.109, and the CV-ANOVA *P*-value was >0.05).

For the targeted analysis, 70 urine metabolites were quantified in the urine of the patients before and after dapagliflozin and insulin degludec treatment as well as of the control group using the Chenomx NMR Suite 8.4 software. At baseline, patients with type 2 diabetes in both dapagliflozin and insulin groups had higher concentrations of 1-methylhistidine, 2-aminobutyrate, 2-hydroxy-3-methylvalerate, 3-chlorotyrosine, 3-hydroxybutyrate, 3-indoxylsulfate, 3-methyl-2-oxovalerate, 3-methyladipate, 3-methylhistidine, 4-hydroxybenzoate, acetoacetate, alanine, betaine, creatine, creatine phosphate, glucose, isoleucine, lactate, leucine, lysine, myoinositol, N-isovaleroylglycine, N-methylhydantoin, N-phenylacetylglycine, phenylalanine, tyrosine, and valerate compared to controls. The levels of butyrate, ethylmalonate, methanol, and sarcosine were significantly lower in these groups compared to those in the control group. We also observed differences in the urine concentrations of some metabolites between the dapagliflozin and insulin groups at baseline. So, the concentrations of 1-methylnicotinamide, 2-hydroxy-3-methylvalerate, 2-hydroxyisobutyrate, 2-hydroxyisovalerate, 2-hydroxyvalerate, 3-hydroxybutyrate, 3-indoxylsulfate, 3-methyl-2-oxovalerate, phenylalanine, and valine were lower in the insulin group compared to the corresponding values in the dapagliflozin group, while that of 2-hydroxybutyrate was higher.

Our results (Table 3) show that 27 of the 70 quantified metabolites were significantly altered by dapagliflozin treatment: 2-aminobutyrate, 2-hydroxy-3-methylvalerate, 2-hydroxybutyrate, 2-hydroxyisovalerate, 3-hydroxybutyrate (+21.2%, *P* < 0.0005), 3-hydroxyisovalerate, acetoacetate (+18.5%, *P* < 0.0005), alanine, betaine (+54.9%, *P* < 0.0001), citrate (+18.4%, *P* < 0.0005), creatine, ethylmalonate, gluconate, glucose (+723.6%, *P* < 0.0001), hippurate, lactate, leucine (+22.2%, *P* < 0.0005), myo-inositol (+77.1%, *P* < 0.0001), N,N-dimethylglycine (+233.8%, *P* < 0.0001), N-methylhydantoin, sarcosine, trigonelline, and valine (+35.1%, *P* < 0.0001) were increased significantly. The metabolites whose concentration was significantly decreased after treatment were 3-chlorotyrosine (−30.5%, *P* < 0.0001), anserine, methanol, and N-isovaleroylglycine. It must be noted that with the exception of 3-hydroxybutyrate, acetoacetate (whose serum concentrations were significantly increased by dapagliflozin), and lactate (whose serum concentrations were significantly decreased by dapagliflozin) all the previously mentioned urine metabolites that were changed by dapagliflozin treatment were either undetectable in the serum or their serum concentration was not modified by the drug (unpublished data). On the other hand, the serum levels of threonine (a metabolite whose urine concentration was not affected by dapagliflozin) was significantly reduced following dapagliflozin administration (from 185.6 ± 52.5 to 167.7 ± 40.6µM, *P* < 0.01).

**Table 3.** Concentrations of Urine metabolites in patients with type 2 diabetes mellitus before and 3 months after administration of dapagliflozin and insulin degludec

Metabolite concentration (μmol/mmol creatinine)	Baseline			Posttreatment				2-way analysis^*a*^	
				Dapagliflozin		Insulin			
	Controls	Dapagliflozin	Insulin	% change	*P*	% change	*P*	*F*	*P*
1-methylnicotinamide	1.97 ± 0.70	2.72 ± 1.70^*^	1.60 ± 0.49^**+**^	−6.6	NS	+15	NS	0.91	NS
1-methylhistidine	9.44 ± 8.24	26.54 ± 14.14^**^	13.18 ± 10.11^*^	+2.4	NS	+17.7	NS	0.14	NS
2-aminobutyrate	4.08 ± 2.02	6.46 ± 2.83^**^	5.85 ± 4.34^**^	+14.4	<0.0005	+6.6	NS	8.92	<0.05
2-hydroxy-3-methylvalerate	3.87 ± 1.67	11.46 ± 6.34^**^	5.56 ± 2.91^*+^	+17.3	<0.0005	+10.2	NS	4.12	<0.05
2-hydroxybutyrate	1.91 ± 1.12	1.20 ± 1.00	3.61 ± 1.64^*+^	+30	<0.0005	+22.2	NS	3.71	<0.05
2-hydroxyisobutyrate	5.66 ± 1.50	8.80 ± 3.08^**^	4.31 ± 2.56^**+**^	−7.6	NS	+5.8	NS	1.61	NS
2-hydroxyisovalerate	1.43 ± 0.59	4.72 ± 1.62^**^	1.79 ± 1.22^**++**^	+14.5	<0.0005	−1.1	NS	4.37	<0.05
2-hydroxyvalerate	2.36 ± 1.33	7.54 ± 6.29^**^	3.33 ± 2.28^**+**^	+2.9	NS	+16.7	NS	0.06	NS
2-oxocaproate	3.58 ± 1.66	2.7 ± 2.03	4.45 ± 3.72	+0.2	NS	−13.1	NS	0.71	NS
3-chlorotyrosine	9.10 ± 12.40	24.17 ± 17.02^**^	20.25 ± 19.27^**^	−30.5	<0.0001	+4.9	NS	3.90	<0.05
3-hydroxybutyrate	12.93 ± 13.10	46.00 ± 41.78^**^	30.46 ± 20.02^**+^	+21.2	<0.0005	−43.1	<0.0005	7.25	<0.05
3-hydroxyisobutyrate	11.12 ± 10.21	12.34 ± 10.96	13.50 ± 12.91	+15.2	NS	−11.1	NS	2.28	NS
3-hydroxyisovalerate	5.16 ± 1.73	4.78 ± 2.83	3.40 ± 2.53^*^	+18	<0.0005	+5.3	NS	3.44	<0.05
3-indoxylsulfate	13.85 ± 8.41	30.6 ± 17.61^**^	21.17 ± 10.35^**+^	−4.4	NS	+11.3	NS	0.69	NS
3-methyl-2-oxovalerate	3.98 ± 2.05	12.23 ± 7.54^**^	9.33 ± 4.88^**+^	−18.4	NS	−10.6	NS	4.32	<0.05
3-methyladipate	2.96 ± 1.85	7.32 ± 6.45^**^	6.65 ± 4.34^**^	+19.1	NS	+8.5	NS	0.92	NS
3-methylhistidine	15.70 ± 8.37	27.16 ± 17.85^**^	22.15 ± 19.22^**^	−1.8	NS	+5.7	NS	0.54	NS
4-hydroxybenzoate	3.14 ± 2.51	5.90 ± 5.76^*^	5.50 ± 3.97^*^	+7.9	NS	+40.1	NS	2.64	NS
Acetate	15.20 ± 11.22	28.07 ± 9.25^*^	20.86 ± 11.68	−1.3	NS	+23.5	NS	1.06	NS
Acetoacetate	14.08 ± 13.53	43.68 ± 16.68^**^	36.05 ± 18.06^**^	+18.5	<0.0005	−45.2	<0.0005	36.68	<0.01
Acetone	4.21 ± 2.07	3.35 ± 2.10	5.49 ± 4.72	+14	NS	−10.7	NS	1.05	NS
Alanine	21.25 ± 9.36	44.63 ± 30.47^**^	40.45 ± 24.93^**^	+26.5	<0.0001	+13.1	NS	3.42	<0.05
Allantoin	10.38 ± 6.83	8.55 ± 5.18	6.64 ± 5.58	+7.2	NS	+7.3	NS	0.25	NS
Anserine	6.57 ± 3.79	9.76 ± 4.51^*^	8.64 ± 6.32	−19.4	<0.0005	−8.3	NS	4.58	<0.05
Betaine	12.96 ± 7.74	72.87 ± 48.04^**^	76.92 ± 42.12^**^	+54.9	<0.0001	−58.3	<0.0005	9.22	<0.01
Butyrate	3.35 ± 1.71	1.69 ± 0.98^*^	1.05 ± 0.84^*^	+15.4	NS	+2.6	NS	1.20	NS
Carnitine	8.12 ± 6.57	6.75 ± 5.85	6.73 ± 7.33	−15.9	NS	−10.4	NS	0.02	NS
Choline	5.68 ± 4.18	6.69 ± 4.78	5.35 ± 4.08	+13.5	NS	+11.2	NS	0.07	NS
cis-Aconitate	22.11 ± 14.96	16.56 ± 8.73	15.27 ± 7.84	−10.9	NS	+9.2	NS	3.02	NS
Citrate	347.10 ± 181.68	357.30 ± 233.27	373.18 ± 273.63	+18.4	<0.0005	−10.5	NS	3.85	<0.05
Creatine	12.62 ± 12.69	49.65 ± 31.64^**^	50.69 ± 37.23^**^	+40.3	<0.0001	+2.3	NS	4.22	<0.05
Creatine phosphate	26.61 ± 18.70	155.92 ± 188.61^**^	143 ± 127.89^**^	+1.6	NS	−6.1	NS	0.04	NS
Cytosine	5.16 ± 3.64	3.35 ± 2.59	6.34 ± 5.58	+1.2	NS	−9.6	NS	0.95	NS
Dimethylamine	35.05 ± 12.24	24.67 ± 10.92^*^	27.23 ± 14.85	+0.1	NS	+18.5	NS	0.68	NS
Ethylmalonate	4.02 ± 1.89	1.64 ± 0.97^**^	1.92 ± 1.34^**^	+33.5	<0.0001	+4.6	NS	6.52	<0.01
Formate	27.89 ± 16.14	22.92 ± 11.72	21.45 ± 21.31	−4.8	NS	+24.2	NS	0.56	NS
Fumarate	0.80 ± 0.56	1.00 ± 0.72	1.23 ± 1.12	−2.0	NS	−14.6	NS	0.66	NS
Gluconate	44.28 ± 18.37	45.55 ± 25.23	37.58 ± 26.88	+33.26	<0.0005	+7.2	NS	5.42	<0.05
Glucose	39.55 ± 14.13	2273 ± 5551^**^	1175 ± 2174^*^	+723.6	<0.0001	−68.1	<0.0005	31.18	<0.01
Glycine	124.18 ± 78.18	121.35 ± 78.74	86.65 ± 76.54	−7.7	NS	+5.8	NS	0.05	NS
Hippurate	325.72 ± 255.79	327.06 ± 259.02	300.01 ± 349.25	+33.3	<0.0005	+7.7	NS	6.33	<0.05
Hypoxanthine	7.59 ± 8.02	5.55 ± 5.05	4.41 ± 4.76	−18.9	NS	+19.5	NS	5.88	<0.05
Indole-3-acetate	6.32 ± 5.03	6.01 ± 4.11	9.35 ± 7.18	−3.3	NS	+8.2	NS	0.65	NS
Indole-3-lactate	7.76 ± 8.11	13.03 ± 6.27^*^	9.35 ± 8.42	−8.5	NS	+5.6	NS	0.58	NS
Isoleucine	2.25 ± 1.01	6.46 ± 3.62^*^	9.88 ± 6.43^**^	+13.3	NS	−9.2	NS	2.85	NS
Kynurenate	2.29 ± 1.66	2.86 ± 2.13	4.17 ± 2.84^*^	+32.1	NS	+28.2	NS	0.74	NS
Lactate	7.94 ± 4.74	12.93 ± 8.43^**^	11.16 ± 7.02^**^	+116	<0.0001	−25.2	<0.0005	9.82	<0.01
Leucine	2.50 ± 1.28	11.27 ± 4.31^**^	9.04 ± 9.03^**^	+22.2	<0.0005	−8.5	NS	4.88	<0.05
Lysine	19.82 ± 15.37	57.45 ± 17.51^**^	46.83 ± 47.27^**^	+7.2	NS	−7.3	NS	1.30	NS
Mannitol	49.68 ± 20.93	72.21 ± 41.17	83.22 ± 117.08	+7.7	NS	−11.3	NS	0.23	NS
Methanol	47.61 ± 30.70	23.31 ± 12.45^**^	16.09 ± 13.63^**^	−22.7	<0.0005	+18	NS	6.10	<0.05
Methylamine	4.79 ± 5.02	5.52 ± 3.86	4.92 ± 4.42	−4.3	NS	−7.11	NS	0.10	NS
Myo-inositol	42.23 ± 26.31	87.61 ± 71.31^**^	82.88 ± 68.71^**^	+77.1	<0.0001	−32.5	<0.0005	14.49	<0.01
N,N-dimethylglycine	2.73 ± 1.38	4.47 ± 3.60	3.54 ± 3.11	+233.8	<0.0001	+1.4	NS	13.92	<0.01
N-isovaleroylglycine	1.37 ± 0.66	3.39 ± 1.91^**^	2.13 ± 1.54^*^	−17.7	<0.0005	+13.6	NS	4.89	<0.05
N-methylhydantoin	10.33 ± 5.45	20.83 ± 9.15^**^	22.44 ± 21.34^**^	+16	<0.0005	+2.9	NS	4.32	<0.05
N-phenylacetylglycine	34.99 ± 13.70	49.64 ± 30.41^*^	58.90 ± 43.36^**^	+13.1	NS	−12.1	NS	4.81	<0.05
Phenylalanine	11.40 ± 12.43	46.72 ± 39.10^**^	30.19 ± 28.36^**+^	−5.2	NS	+38.1	NS	2.25	NS
Pyruvate	4.40 ± 2.52	5.56 ± 3.17	5.05 ± 3.20	−3.9	NS	−3.9	NS	0.06	NS
Sarcosine	6.07 ± 7.46	1.66 ± 0.88^**^	1.98 ± 1.53^**^	+109	<0.0005	−14.1	NS	56.31	<0.01
Succinate	5.29 ± 3.39	12.49 ± 5.89	13.99 ± 12.4^*^	+5.8	NS	−2.3	NS	0.03	NS
Threonine	21.59 ± 10.92	36.61 ± 21.63^*^	29.20 ± 22.91	+10.4	NS	+10.5	NS	0.01	NS
trans-Aconitate	5.26 ± 4.18	4.24 ± 3,81	2.94 ± 1.91	−24.5	NS	+0.7	NS	2.41	NS
Trigonelline	33.46 ± 35.39	35.69 ± 28.77	43.02 ± 19.15	+96.1	<0.0001	+7.6	NS	22.37	<0.01
Trimethylamine	2.08 ± 0.96	3.79 ± 1.91	4.34 ± 4.13	−2.6	NS	+3.7	NS	0.22	NS
Trimethylamine N-oxide	50.40 ± 41.32	36.38 ± 37.80	36.63 ± 28.06	−16.8	NS	+2.9	NS	0.47	NS
Tyrosine	6.78 ± 3.82	19.11 ± 7.38^**^	15.81 ± 14.69^**^	+9.4	NS	−2.1	NS	0.29	NS
Valerate	4.69 ± 3.20	20.96 ± 11.73^**^	15.95 ± 13.00^**^	−28.72	NS	+1.2	NS	1.99	NS
Valine	3.63 ± 1.28	8.56 ± 4.10^**^	3.11 ± 1.91^**++**^	+35.1	<0.0001	+19.2	NS	4.45	<0.05
Xanthurenate	9.81 ± 10.26	18.17 ± 12.75^*^	12.15 ± 28.24	+11.8	NS	+28.2	NS	1.23	NS

^*a*^ *F*- and *P*-values for the treatment*time point interaction.

^*^ *P* < 0.0005 and ^**^P < 0.0001 compared to control group baseline values.

^**+**^ *P* < 0.0005 and ^**++**^*P* < 0.0001 compared to dapagliflozin group baseline values.

The results in the targeted analysis are nearly totally consistent with those found in the untargeted multivariate analysis. With both methods, all metabolites are altered to the same direction and most of them with similar significance. We observed discrepancy only in 2 metabolites, TMAO and N,N-dimethylglycine, due to the high values of SD.

The increases in the urinary excretion of the BCAAs were significantly correlated with the corresponding increase in the degree of glycosuria after dapagliflozin administration (Pearson correlation coefficients 0.346, 0.339, and 0.467 for leucine, isoleucine, and valine, respectively; *P* < 0.05 for all correlations). In addition, the baseline values of betaine and myo-inositol were highly correlated with urine concentration of glucose (Pearson correlation coefficients 0.511 and 0.658 for betaine and myo-inositol, respectively; *P* < 0.001 for all correlations) whereas the increase in the urine concentrations of these compounds following dapagliflozin administration was also significantly correlated with the drug-induced increase in glycosuria (Pearson correlation coefficients 0.341 and 0.397 for betaine and myo-inositol, respectively; *P* < 0.001 for all correlations).

Although insulin degludec improved glycemia to a degree similar to that observed with dapagliflozin, it had no effect on the levels of the majority of urine metabolites. In addition to a reduction in glycosuria, insulin degludec reduced the urine concentrations of 3-hydroxybutyrate, acetoacetate, betaine, lactate, and myo-inositol (Table 3). Two-way analysis for time points revealed that all the statistically significant dapagliflozin-induced changes in urine metabolites were also different from the effect of insulin on the levels of these metabolites (Table 3). In addition, although the changes in the concentrations of 3-methyl-2-oxovalerate (−18.4%), hypoxanthine (−18.9%), and N-phenylacetylglycine (+13.1%) after dapagliflozin treatment did not reach statistical significance, these alterations were found to be significantly different from those observed after insulin administration by 2-way analysis.

## Discussion

In the current study, we show that patients with type 2 diabetes exhibit an altered urine metabolic profile characterized by changes in the concentrations of ketone bodies, osmolytes, amino acids, and various other metabolites. There were differences in the values of some metabolites between the patients with type 2 diabetes included in the dapagliflozin and insulin groups at baseline; however, these can be attributed to the lack of randomization. Although most of the clinical and conventional laboratory characteristics of the 2 groups were similar, residual confounding due to unmeasured factors cannot be excluded.

Along with the expected increase in glycosuria, treatment with dapagliflozin resulted in significant changes in the renal excretion of amino acids and their derivatives, tricarboxylic acid cycle intermediates, amines, organic acids, and products of gut microbial origin.

In agreement with previous studies, we found a significant increase in the urine concentrations of ketone bodies (3-hydroxybutyrate and acetoacetate) as well as lactate following dapagliflozin administration (17). However, whether these changes represent drug-induced modifications in the tubular handling of these compounds, reflect shifts in the energy metabolism of renal cells, or result from the increased systemic production of these compounds remains indeterminate. On the other hand, insulin significantly reduced the urine concentrations of these metabolites, a finding that can be attributed to parallel changes in the serum concentrations of these compounds and/or to changes in their renal metabolism and tubular handling.

The essential BCAAs leucine, isoleucine, and valine are important for tissue expansion and regeneration and are involved in various metabolic functions. BCAAs are found in abundance in dietary proteins, and it has been shown that a BCAA-rich diet correlates positively with metabolic health, including regulation of body weight, muscle protein synthesis, and glucose homeostasis. However, cross-sectional and prospective human studies have highlighted that increased fasting concentrations of circulating BCAAs and BCAA supplementation are associated with an increased risk for insulin resistance and type 2 diabetes (18). So, the effects of BCAA on human metabolism can be considered as pleiotropic, depending on host metabolic state. BCAA catabolism involves 2 steps: a reversible one—catalyzed by a branched-chain aminotransferase (BCAT), either cytosolic or mitochondrial, requiring pyridoxal to function as an amino group carrier by which the BCAA and 2-ketoglutarate produce a branched-chain keto acid and glutamate, and the irreversible mitochondrial process catalyzed by branched-chain keto acid dehydrogenase (BCKDH) leading to the formation of acetyl-CoA, propionyl-CoA, and 2-methyl-3-hydroxybutyryl-CoA (from leucine, valine, and isoleucine, respectively), which enter the tricarboxylic acid cycle leading to adenosine 5′-triphosphate formation (19). Impaired function of the BCAT and BCKDH enzymes has been observed in genetic disorders such as maple syrup urine disease or as a result of elevated concentrations of fatty acids, proinflammatory cytokines, or insulin. The resulting accumulation of branched-chain keto acids and other metabolites potentially contributes to the development of insulin resistance and decreases further the expression of BCAT and BCKDH in skeletal muscles, thus leading to the establishment of a vicious cycle that predisposes to the development of type 2 diabetes (19). Our results suggest that dapagliflozin increases the urine concentration of BCAAs. These effects presumably result from the decreased proximal reabsorption of these metabolites since dapagliflozin increased neither their serum levels nor eGFR. The mechanism and the clinical significance of this phenomenon remain indeterminate. However, previous studies have shown that proximal tubular cells in culture incorporate leucine more readily when they are incubated in media with high concentrations of glucose or sodium (20). This process possibly involves internalization of glucose and sodium through SGLT-2 and Na(+)/H(+) exchanger (NHE3) transporters (21-23). As a consequence, we propose that the inhibition of SGLT-2 and possibly NHE3 transporters by dapagliflozin is responsible for the increased renal excretion of BCAAs. Indeed, the percentage changes in the excretion rates of all 3 BCAAs showed a significant correlation with the changes in glucose excretion. Since the incorporation of amino acids is an essential step for proximal tubular cell hypertrophy (an early manifestation of diabetic nephropathy), it can be assumed that the dapagliflozin-induced decrease in the reabsorption of BCAAs may contribute to the regression of proximal tubular hypertrophy (24).

In the present study, we identified 6 BCAA catabolism intermediates in the urine of patients with type 2 diabetes (Supplementary Figure 5A (16)). More specifically, 2-hydroxy-3-methylvalerate, 2-hydroxyisovalerate, and 3-hydroxyisovalerate were significantly increased after dapagliflozin administration whereas 3-hydroxyisobutyrate fell short of statistical significance probably due to a large SD value. In addition, 2 toxic metabolites—namely, 3-methyl-2-oxovalerate and N-isovaleroylglycine (arising from the incomplete breakdown of isoleucine and leucine, respectively) that were found to be increased in patients with type 2 diabetes compared to controls—were significantly decreased. These changes possibly reflect an improvement in BCAA metabolism and are in line with those observed after empagliflozin administration (12).

Another novel finding of our study is the almost 2-fold increase in the urine concentrations of betaine and myo-inositol after dapagliflozin administration. These “compatible organic osmolytes” are present in high concentrations in renal medulla where they protect renal cells from hypertonicity (25). The increased urine levels of these molecules following dapagliflozin treatment may result from a decrease in their tubular reabsorption or may represent an adaptive response to the increased medullary tonicity that results from massive glycosuria and natriuresis. Both betaine and myo-inositol urine concentrations showed a significant correlation with glucose and sodium excretion at baseline. In addition, the increase in their urine concentrations following dapagliflozin administration significantly correlated with the changes in the concentration of glucose. The reduction in the urine concentrations of betaine and myo-inositol with insulin degludec, which reduced significantly the degree of glycosuria, is in line with this assumption.

In addition to their role as osmolytes, betaine and myo-inositol may also exert additional beneficial effects that possibly contribute to the renoprotective properties of dapagliflozin. Indeed, previous studies have shown that myo-inositol protects renal cells during their exposure to high glucose concentrations and reduces the fibrotic changes that characterize the development of diabetic nephropathy (26). Further, myo-inositol depletion has been shown to exert deleterious effects on tubular cells (27). This depletion may result from glucose toxicity since high glucose concentrations upregulate the myo-inositol-degrading enzyme myo-inositol oxygenase (28). In this perspective, the dapagliflozin-induced increase in urine myo-inositol may reflect the reduction in the exposure of tubular cells to high glucose concentrations and the restoration of cell metabolism. Similarly, previous studies suggest that betaine has important anti-inflammatory actions (29), reduces liver steatosis (30) and improves mitochondrial content and function in liver cells (31). Interestingly, a recent metabolomic study in patients with type 2 diabetes treated with dapagliflozin identified changes in urine metabolites consistent with an improvement in mitochondrial function (13). Whether increased renal concentrations of betaine contribute to this effect remains to be established.

N-methylhydantoin is an oxidative metabolite of creatinine. Previous studies in cell lines and animal models of kidney injury revealed that this compound exerts important antioxidant properties and can protect tubular cells during their exposure to various toxic insults (32,33). We found that dapagliflozin induced a significant increase in the urine concentration of N-methylhydantoin. Since N-methylhydantoin is produced by the metabolism of creatinine by gut microbes it appears tempting to hypothesize that the previously described effect of the drug on gut microbiota (34) may contribute to the observed increase in the renal excretion of this compound. On the other hand, alterations in the renal handling of N-methylhydantoin may also play a role in these changes. The microbial decomposition of creatinine can also proceed via creatine as the first degradation product. Both N-methylhydantoin and creatine are further metabolized to sarcosine (Supplementary Figure 5B (16)). Sarcosine can also be produced by choline that escapes microbial degradation and is oxidized to betaine, which is converted to N,N-dimethylglycine and then to sarcosine. This pathway is important for osmoregulation and as a source of methyl groups (35). In our study, the excretion of the previously mentioned metabolites N-methylhydantoin, creatine, betaine, sarcosine, and N,N-dimethylglycine was increased after dapagliflozin treatment, a finding suggesting that the drug may have a beneficial effect on gut microbiota metabolism as previously reported (36).

Methylglyoxal, a highly reactive dicarbonyl aldehyde, is a major precursor of nonenzymatic glycation of proteins and DNA, leading to the formation of advanced glycation endproducts that can affect the function and structure of organs and tissues and has subsequently been implicated in the pathogenesis of type 2 diabetes and its complications (37). Methylglyoxal is a by-product of glycolysis but can also be produced by the catabolism of threonine (Supplementary Figure 5C) (16,37). The principal threonine catabolic pathway in humans involves a glycine-independent serine/threonine dehydratase yielding 2-ketobutyrate, which is further catabolized to propionyl coenzyme A (CoA) and then to succinyl CoA or to 2-aminobutyrate and 2-hydroxybutyrate. Threonine can also be converted to 2-amino-3-ketobutyrate and then to glycine and acetyl-CoA. Alternatively, 2-amino-3-ketobutyrate can be decarboxylated nonenzymatically to aminoacetone and then to pyruvate or to methylglyoxal (38). In our study, succinate and pyruvate were not increased but 2-aminobutyrate and 2-hydroxybutyrate were significantly increased. These findings along with the decrease in the serum levels of threonine in our study following dapagliflozin treatment indicate an altered catabolism of threonine that does not favor the formation of methylglyoxal. In addition, the dapagliflozin-induced decrease in glucose utilization for energy production may further results in a decrease in the concentrations of methylglyoxal, which, in turn, may translate into a reduction in the complications of diabetes. However, how dapagliflozin affects threonine metabolism remains indeterminate.

The renal excretion of the compounds related to muscle metabolism, alanine and anserine, were also significantly altered by dapagliflozin administration. Alanine, a nonessential amino acid, is highly concentrated in muscles, functioning as key in glucose-alanine cycle between tissues and liver. Its biosynthesis occurs either from the conversion of pyruvate or the breakdown of DNA and the dipeptides carnosine and anserine. In our study, urine alanine concentration was significantly higher following dapagliflozin therapy. This change can be attributed either to the increased concentrations of this metabolite systemically or locally or to its decreased tubular reabsorption (39). In tubular cells alanine can be used either for ammoniagenesis, gluconeogenesis, or energy production (40,41). Dapagliflozin, by inhibiting NHE3 exchangers in proximal tubular cells may decrease net acid excretion and thus the use of alanine as ammonia precursor. This, in turn, may result in increased concentration of this amino acid in tubular cells and increased urine leakage. On the other hand, the use of alanine as a substrate for gluconeogenesis in renal cells following dapagliflozin treatment has not been determined. Previous studies provided conflicting data on the effect of SGLT-2 inhibitors on endogenous glucose production (42-44), whereas the role of the kidney in this process remains elusive. Although the clinical significance of the increased urine concentrations of alanine after dapagliflozin administrations is unknown, experimental studies suggest that alanine may protect tubular cells form hypoxic injury (45).

Trigonelline, N-methyl nicotinic acid or betaine nicotinate, is a product of niacin (vitamin B3) metabolism that is excreted in the urine. Experimental studies have shown that trigonelline ameliorates diabetic hypertensive nephropathy by suppression of oxidative stress in kidney and reduction in renal cell apoptosis and fibrosis (46). Trigonelline is also reported as a constituent found in tissues that correlates positively with lean mass quantity of which the physiological properties remain unexplored and also as a metabolite that could reflect part of the metabolism of choline through betaine and glycine metabolic pathways (47). The statistically significant increased levels in our study after dapagliflozin treatment may indicate an improved metabolic status of our patients.

Proximal tubules have a very high content of mitochondria and are highly dependent on oxidative phosphorylation. Previous studies in diabetic and nondiabetic models of chronic kidney disease revealed significant disruption of mitochondrial function that parallels the evolution of the disease and is translated into abnormal renal excretion of citric acid cycle (tricarboxylic acid) intermediates (48,49). In our patients, we observed a significant increase in the renal excretion of citrate following dapagliflozin treatment, a finding that may indicate a restoration of mitochondrial function and an improvement in energy metabolism in tubular cells. Other conditions that may increase citrate excretion like potassium depletion, acid-base abnormalities, hyperparathyroidism, or protein-rich diet are unlikely in our population. Hippurate is a metabolite normally found in human urine. It is synthesized in the kidney and liver from glycine and benzoic acid, secreted by the renal tubular cells, and excreted in the urine. Dapagliflozin altered hippurate excretion in the same direction as did for citrate. This covariation that has been also noted in certain renal disorders can be explained by the link between hippurate synthesis and mitochondrial function. The first step in the formation of hippurate from benzoate in the mitochondrial matrix requires adenosine 5′-triphosphate. Thus, it is possible that impaired mitochondrial functioning may have contributed to the lower levels of hippurate excretion before treatment (50).

We also observed altered levels of a number of less studied metabolites such as ethylmalonate, nutrition-related gluconate, and 3-Chlorotyrosine. Ethylmalonate, also known as alpha-carboxybutyric acid, is a breakdown product of butyrate and member of the class of compounds known as branched fatty acids. Ethylmalonate may reflect metabolic processes involved in long-chain fatty acid metabolism (such as carnitine-dependent pathways) and related mitochondrial function, and it is mainly studied in cases of *i*nherited metabolic disorders (51).

3-chlorotyrosine, a specific marker of myeloperoxidase-catalyzed oxidation, was decreased after treatment in our study. It has been reported that increased levels of 3-chlorotyrosine predict chronic kidney disease severity and associated coronary artery disease (52).

Our study has limitations. The most important of them is probably the lack of a placebo arm. Although the inclusion of a control group of age- and sex-matched healthy individuals allows the interpretation of the direction of the changes in the urine concentrations of the various metabolites, the precise estimation of their magnitude remains problematic. On the other hand, the inclusion of an insulin arm excludes the possibility that the dapagliflozin-induced changes in urine metabolome are the consequence of the improvement in glycemia, a conclusion that could not have been drawn had a placebo group been utilized. We understand that the mechanisms we propose for the observed dapagliflozin-induced changes in urine metabolome are speculative. However, since metabolomic studies usually detect changes in the concentrations of several metabolites, the complete characterization of the underlying mechanisms and the potential clinical consequences is extremely difficult. We tried to base our speculations on solid scientific evidence, but we believe that further studies are required to confirm and extend our observations.

In conclusion, our results suggest that dapagliflozin treatment is associated with a significant change in urine metabolome. On a pathophysiological basis, most of the observed changes in the urine concentrations of the individual metabolites can be considered beneficial and may contribute to the renoprotective properties of dapagliflozin. Further studies are needed to confirm our results and to explore the mechanisms that underlie them.

## Data Availability

Data sets generated during and/or analyzed during the current study are not publicly available but are available from the corresponding author on reasonable request.

## Abbreviations

BCAAs: branched-chain amino acids
BCAT: branched-chain aminotransferase
BCKDH: branched-chain keto acid dehydrogenase
FE: fractional excretion
NHE3-transporters: Na(+)/H(+) exchanger
^1^H-NMR: proton-nuclear magnetic resonance
OPLS-DA: orthogonal projections to latent structures discriminant analysis
PCA: principal component analysis
SGLT-2: sodium glucose co-transporters-2
TSP: sodium 3-trimethylsilyl (2,2,3,3,-2h4) propionate.

## Additional Information

***Disclosures:*** Authors have nothing to declare
